# Serum cotinine as a predictor of lipid-related indices in Turkish immigrants with type 2 diabetes: A clinic-based cross-sectional study

**DOI:** 10.3389/fmed.2023.1011045

**Published:** 2023-02-16

**Authors:** Shiryn D. Sukhram, Gustavo G. Zarini, Lemia H. Shaban, Joan A. Vaccaro, Avinash R. Sukhram, Fatma G. Huffman

**Affiliations:** ^1^Department of Biology, College of Staten Island, City University of New York, New York City, NY, United States; ^2^Clinical & Scientific Research, Oxford Biomedical Technologies, West Palm Beach, FL, United States; ^3^Department of Food Science and Nutrition, College of Life Sciences, Kuwait City, Kuwait; ^4^Department of Dietetics and Nutrition, Robert Stempel College of Public Health and Social Work, Florida International University, Miami, FL, United States; ^5^Kymera Hospitalist Group, Naples, FL, United States

**Keywords:** cardiovascular disease, cotinine, diabetes, immigrants, lipids, neighbourhood deprivation, smoking

## Abstract

**Background:**

Turkish immigrants form the largest ethnic minority group in the Netherlands and show a higher prevalence of (i) cardiovascular disease (CVD), (ii) cigarette smoking, and (iii) type 2 diabetes (T2D) as compared to the native Dutch. This study examines the association of CVD risk factors: serum cotinine, as an indicator of cigarette smoke, and lipid-related indices among first-generation (foreign-born) Turkish immigrants with T2D living in deprived neighbourhoods in the Netherlands.

**Methods:**

A total of 110 participants, physician-diagnosed with T2D, aged 30 years and older, were recruited by convenience sampling from the Schilderswijk neighbourhood of The Hague in a clinic-based cross-sectional design. Serum cotinine (independent variable) was measured with a solid-phase competitive chemiluminescent immunoassay. Serum lipids/lipoproteins (dependent variables) were determined by enzymatic assays and included: total cholesterol (CHOL), high-density lipoprotein cholesterol (HDL-c), low-density lipoprotein cholesterol (LDL-c), and triglycerides (TG). The Castelli Risk Index-I (CRI-I), and Atherogenic Coefficient (AC) were calculated using standardised formulas and assessed as dependent variables in multiple linear regression (MLR) models. Log-transformation of HDL-c, TG, CRI-I, and AC values were performed to account for the extreme right skewness of the data. Statistical analyses included descriptive characteristics and MLR models were adjusted for all major confounders of cotinine and lipids.

**Results:**

The sample size had a mean age of 52.5 years [standard deviation (SD) = 9.21]. The geometric mean of serum cotinine level was 236.63 ng/mL [confidence interval (CI) = 175.89 ± 318.36]. The MLR models indicated that high serum cotinine levels (≥10 ng/mL) was positively associated with HDL-c (*P* = 0.04), CRI-I (*P* = 0.03), and AC (*P* = 0.03) in the age, gender, WC, diabetes medications, and statins-adjusted models (*n* = 32).

**Conclusion:**

This study indicated that lipid ratios of HDL-c, CRI-I and AC are dependent determinants of serum cotinine and higher serum cotinine levels (≥10 ng/mL) are associated with worse HDL-c, CRI-I and AC values in participants with T2D. Clinical comprehension of these biochemical indicators (lipids/lipoproteins) and symptomatic results (CVD risk) in individuals with T2D will aid in the intervention (smoking) approach for this vulnerable cohort (Turkish immigrants). Therapy that is targetted to modify this behavioural risk factor may improve cardiovascular health outcomes and prevent comorbidities in Turkish immigrants with T2D living in deprived neighbourhoods in the Netherlands. In the meantime, this report contributes to a growing body of information and provides essential guidance to researchers and clinicians.

## Introduction

Type 2 diabetes (T2D) is highly prevalent among ethnic minorities living in Western societies. Comparable to other Western countries such as the United Kingdom and the United States of America (USA) (1–3) the overall cardiovascular mortality is typically higher among ethnic minority groups as compared to the general population of the Netherlands (4). In the Netherlands, Turkish immigrants form the largest ethnic minority group with 429,978 inhabitants according to 2022 census (5). Epidemiological studies report that people living in deprived neighbourhoods have an increased risk for developing cardiovascular diseases (CVD) as compared to the general population (6). The Hague, Netherlands, a city that is part of the Randstad metropolitan area, is vastly urbanised with a large population of Turkish immigrants (*n* = 75,423). The Schilderswijk neighbourhood is considered a deprived area of The Hague populated by 91.8% non-Western immigrants and 8.2% native Dutch (7, 8). According to Gemeente Den Haag census, Turkish immigrants are the most prevalent inhabitants of the Schilderswijk area (*n* = 4,005) (8). Furthermore, 61.3% residents in the Schilderswijk area are aged 20–64 years, with an average age of 35 years (8). Kriegsman et al. (9) indicate that the onset of diabetes occurs one decade earlier among the Turkish when compared to the native Dutch. Moreover, scientific literature identifies mortality from CVD in patients with T2D to be higher than in the general population (10, 11).

Diabetes, cigarette smoking, and dyslipidaemia are well-known risk factors for CVD in the general population in the Netherlands (12). Compared to the native Dutch population, Turkish immigrants in the Netherlands show a higher prevalence of (i) CVD, (ii) cigarette smoking, and (iii) T2D (9, 13–16). However, El Fakiri et al. (17) indicate that ethnic minorities are less likely to smoke as compared to the native Dutch (OR = 0.10–0.53). Yet, data from the Lifestyle in Amsterdam: Study among Ethnic gRoups (LASER)-study (16) report a higher prevalence of smoking in first-generation Turkish males and second-generation Turkish females as compared to native Dutch males and females. Hosper et al. (16) suggest that the smoking prevalence rates of second-generation Turkish males are comparable to the prevalence rates among Dutch males. Recently, Jain and Ducatman (18) analysed data from the National Health and Nutrition Examination Survey (NHANES) for the period 1999–2012 to examine the effect of serum cotinine (<10 ng/ml classified as non-smokers, ≥10 ng/mL classified as smokers) on the levels of total cholesterol (CHOL), high-density lipoprotein cholesterol (HDL-c), low-density lipoprotein cholesterol (LDL-c), and triglycerides (TG) for the US population aged ≥20 years. Findings of the aforementioned study suggest that serum cotinine is significantly associated with unfavourable lipid/lipoprotein profiles among the adult population of the USA. The clinical use of serum cotinine, rather than self-reported smoking status, in epidemiological studies has added to our understanding of the association between cigarette smoking and adverse lipid profiles. It is important to examine molecular biomarkers of modifiable health risks among high-risk populations and serum cotinine may be a better indicator of quantifying risks from cigarette use as opposed to self-reports (19).

Research indicates that Turkish immigrants in the Netherlands are 2.4 times more likely to be obese, but have a lower prevalence of hypercholesterolaemia (17). According to the 2002 Rijksinstituut voor Volksgezondheid en Milieu (RIVM) report (13), 80% of Turkish immigrants aged 35 years or older are overweight. A cross-sectional study done among Turkish and Moroccan migrants in the Netherlands, show that specific subpopulations are at particular risk for overweight and obesity and ethnic-specific interventions should address all first-generation immigrants (20). Little is known about the association of serum cotinine and lipid abnormalities among first-generation Turkish immigrants with T2D. Considering the high prevalence of smokers among the Turkish population in the Netherlands (16), it is more than likely that a significant amount of the population who do not smoke may be exposed to second hand smoke exposure (SHS). Additionally, the increased prevalence of smoking among first-generation Turkish immigrants may be related to external stressors such as being diagnosed with T2D and/or living in deprived neighbourhoods. Therefore, the objective of this study was to examine the relationship between serum cotinine, as an indicator of cigarette smoke, with lipid-related indices among first-generation Turkish immigrants with T2D living in deprived neighbourhoods of The Hague, Netherlands.

## Materials and methods

### Participants

The study population consist of 110 first-generation Turkish immigrants, physician-diagnosed with T2D, aged 30 years and older. First-generation Turkish immigrants were defined according to the definition of Statistics Netherlands for non-Dutch first-generation: born outside the Netherlands with one or both parents born outside the Netherlands (21, 22). All participants were diagnosed with T2D as recommended by the 2006 Nederlands Huisartsen Genootschap (NHG) standard and/or use of diabetes medication (23).

### Sampling

The investigation took place from March to May 2011 in a clinic-based cross-sectional design. Turkish immigrants were recruited from the deprived Schilderswijk area of The Hague. Neighbourhood deprivation in the Netherlands was determined according to a validated index (7). Supplementary Figure 1 illustrates the convenience sampling design used for participant recruitment in this non-randomised cross-sectional study. During the 3-month period, 300 letters written in Dutch and Turkish languages outlining the study were mailed to residents of the Schilderswijk area with Turkish surnames listed in the local telephone directory. The interested participants could respond to the invitation letter. Due to unknown addresses, 1% of the unopened letters were returned undeliverable. Posters and flyers were also displayed at general practitioner (GP) offices, dieticians’ offices, health club centres, mosques, hair salons, Turkish grocery stores, community centres, and pharmacies where Turkish immigrants were known to frequently visit. Local GP’s actively told their patients about, and referred them to, our research study. From this, 11 eligible participants were enrolled from the delivered mail, 60 participants were enrolled from GP offices, and 20 participants from posters and flyers. Recruitment of participants through word of mouth was actively promoted by a Turkish community representative in the Schilderswijk area, where participants of the study were encouraged to recruit other participants (*n* = 19 eligible participants). Twenty-two potential participants did not qualify for the study because they were either not Turkish (*n* = 3), did not have T2D (*n* = 8), or did not provide blood samples within the 3-month study period (*n* = 11). Interested participants were initially interviewed on the phone, at which time the study purpose was explained, and age and gender of the responders were recorded.

### Physical exam

A research assistant (RA) fluent in both Dutch and Turkish languages interviewed participants in their preferred language. The RA assisted and completed questionnaires on age, education, gender, Islamic diet, marital status, parity, and prescription medication(s) use including diabetes medications and statins. Parity number was defined as the number of live childbirth (number of live birth) plus the number of stillbirth (defined as birth of an infant that died after the 20th week of pregnancy in the uterus).

Blood pressure (BP) was measured by clinical protocol using an automated sphygmomanometer with the participant resting alone in a quiet room. Measurements were performed twice and the average reading taken as the participant’s BP. The BP readings were displayed in pairs, with the upper (systolic) value first, followed by the lower (diastolic) value. Waist circumference (WC) to the nearest 0.1 cm was measured horizontally with a non-stretchable measuring tape placed midway between the 12th rib and iliac crest at minimal respiration to measure central obesity. Body mass index (BMI) was calculated as weight (kg) divided by height (m^2^).

The Department of Phlebotomy at Haaglanden Medisch Centrum (HMC) Westeinde collected participants’ serum samples during physical exams, aliquoted them, and kept them frozen at −20°C until they could be analysed. Venous blood (20 mL) was collected after an 8–12 h overnight fast, by a certified phlebotomist who used standard laboratory techniques. Serum measurements were performed at HMC hospital laboratory.

### Independent variable: Cutoff of serum cotinine level (≥10 ng/mL)

Cotinine, the primary metabolite of nicotine, is a prognostic biomarker used to differentiate smokers from non-smokers in epidemiologic studies. Because of its longer half-life, cotinine, measured in serum, urine, or saliva, allows for differentiating active smokers from passive and non-smokers (24). According to the Centers for Disease Control and Prevention (CDC)–National Biomonitoring Programme (25), non-smokers exposed to SHS have serum cotinine levels <1 ng/mL, with high SHS exposure yielding values in the 1–10 ng/mL range. High serum cotinine levels (≥10 ng/mL) indicate active smokers. Serum cotinine was measured with a solid-phase competitive chemiluminescent immunoassay.

### Dependent variables: Lipid/lipoprotein-related indices

Diabetic dyslipidaemia is commonly illustrated by high CHOL, low HDL-c, high TG, and high LDL-c (26). It is widely recognised that these lipid variations characterise the major association between T2D and increased cardiovascular risk of diabetic patients. Smoking has also been shown to alter lipid/lipoprotein levels (18). Serum lipids and lipoproteins were determined by enzymatic *in vitro* assay and included: CHOL, HDL-c, LDL-c, and TG.

### CVD risk measurements

The Castelli risk index-I (CRI-I), also known as cardiac risk ratio (CRR), suggests the development of coronary plaques with a diagnostic value as good as the determination of CHOL (27). The CRI-I was calculated by the standardised formula: CRI-I = CHOL/HDL-c. The scientific literature report that in an effort to enhance the predictive capacity of lipid-related indices, several lipoprotein ratios or “atherogenic indices” have been defined. The atherogenic coefficient (AC) is a significant index that can be used as a stand-alone index for CVD risk estimation (28). The AC was calculated by the standardised formula: AC = (CHOL–HDL-c)/HDL-c.

### Covariates

To control for potential confounding between serum cotinine level and lipid-related indices, after reviewing several related studies and based on clinical significance of factors that affect the independent and/or dependent variables (18), we included the following covariates in the analysis- age (years), gender (male or female), waist circumference, education (some school or high school graduate or above), diabetes medications (yes or no), and statins (yes or no). It has been established that there are differences in lipids by gender and age. Percent of fat, of which WC is a proxy variable, is associated with lipids and lipid markers. We therefore included WC, instead of BMI, since it is proven to be a better indicator of body fat around the visceral organs which is associated with metabolic complications. The aforementioned covariates are also associated with glycated haemoglobin (HbA1c) values, which is clinically considered to be the gold standard for monitoring glycaemic control in patients with T2D. All the above information was collected from face-to-face interviews or assessed during physical exams.

### Statistical analyses

Serum cotinine level was analysed as a continuous variable. There were no missing values in the study sample (*n* = 110). Statistical analyses were performed on participants with complete data on all variables required for multiple linear regression (MLR) models. Differences between two categories of cotinine (high ≥10 ng/mL and low <10 ng/mL) were assessed using the independent samples *t*-test for numerical values and the chi-square test for categorical variables. The dependent variables were checked for normal distribution using the Shapiro-Wilks Normality Test, and homogeneity of variance using the Breusch-Pagan/Cook-Weisberg test for heteroskedasticity. Log-transformation of HDL-c, TG, CRI-I, and AC values were performed to account for the skewed nature of the raw data. All dependent variables conform to homogenous variance (*P* < 0.10). We ran MLR models to calculate the mean change in CHOL, HDL-c [log], LDL-c, TG [log], CRI-I [log], and AC [log] separately, for high serum cotinine levels (≥10 ng/mL) as a continuous variable (*n* = 32). Participants with low serum cotinine levels (<10 ng/mL) had values of 0 ng/mL (*n* = 78). We therefore categorised high serum cotinine level into quartiles. We ran MLR models to calculate the mean change in CHOL, HDL-c [log], LDL-c, TG [log], CRI-I [log], and AC [log] for each higher serum cotinine level by taking the lowest category as the referent (10≥cotinine ≤199 ng/ml). All MLR models were adjusted for age, gender, WC, diabetes medications, and statins. Stata 10.1 (StataCorp LP, College Station, TX) was used for statistical analysis and *P* < 0.05 was considered significant.

## Results

Table 1 presents the baseline characteristics of the study population, all of whom were physician-diagnosed with T2D. There were no missing values for the participants therefore none were excluded from analysis. The 110 participants that were included in the study had a mean age of 52.5 years [standard deviation (SD) = 9.21]. Among the 32 participants that had high cotinine levels (≥10 ng/mL), approximately half of them were female (46.9%), married (68.8%), completed some high school or above (90.6%), followed an Islamic diet (93.8%), had a BMI >30 kg/m^2^ (59.4%), and were taking statins (53.1%). The 32 participants, with high cotinine levels (≥10 ng/mL), had a mean of 1.75 of parity, had a mean of 103.63 cm of waist circumference, a mean of 139.5 mmHg systolic blood pressure, a mean of 82.53 mmHg diastolic blood pressure, a mean of 4.99 mmol/L CHOL, a mean of 0.08 mmol/L HDL-c [log], a mean of 3.09 mmol/L LDL-c, a mean of 0.56 mmol/L TG [log], a mean of 1.49 CRI-I [log], a mean of 1.21 AC [log], and a mean of 51.75 mmol/L HbA1c. Education, HDL-c [log], CRI-I [log], and AC [log] were statistically significant with high cotinine levels (≥10 ng/mL): *P* = 0.02, *P* = 0.04, *P* = 0.02, and *P* = 0.02, respectively. Parity had a weak association with high cotinine levels (*P* = 0.05). The geometric mean of serum cotinine level was 236.63 ng/mL [confidence interval (CI) = 175.89 ± 318.36].

**TABLE 1:** Baseline characteristics of study participants by serum cotinine cutoff of ≥10 ng/mL.

	Cotinine (ng/mL)		
Variables	High^a^ (*n* = 32)	low^b^ (*n* = 78)	*P*
Age (years)	50.66 ± 7.38	53.96 ± 9.74	0.09
Gender (F) (%)	46.88	62.82	0.12
Marital status (Yes) (%)	68.75	78.21	0.3
Education (Yes) (%)	90.63	69.23	**0.02** *
Parity	1.75 ± 2	2.71 ± 2.47	0.05
Islamic diet (Yes) (%)	93.75	87.18	0.32
BMI (>30 kg/m2) (%)	59.38	67.95	0.39
WC (cm)	103.63 ± 9.36	105.60 ± 10.93	0.37
HbAlc (mmol/L)	51.75 ± 14.35	51.73 ± 14.94	0.99
SBP (mmHg)	139.5 ± 23.96	142.58 ± 20.23	0.49
DBP (mmHg)	82.53 ± 9.27	82.92 ± 10.28	0.85
CHOL (mmol/L)	4.99 ± 1.24	4.75 ± 1.12	0.32
HDL-c (mmol/L) [Log]	0.08 ± 0.27	0.2 ± 0.27	**0.04** *
LDL-c (mmol/L)	3.00 ± 1.08	2.73 ± 0.94	0.08
TG (mmol/L) [log]	0.57 ± 0.45	0.49 ± 0.51	0.48
CRI-I [log]	1.49 ± 0.34	1.33 ± 0.32	**0.02** *
AC [log]	1.22 ± 0.45	1 ± 0.47	**0.02** *
Statins (yes) (%)	53.13	50	0.77

Data are means ± SD unless otherwise indicated.

*P is considered significant at <0.05. Bolded values mean statistically significant results.

^a^High cotinine is defined as ≥10 ng/mL. Level for cotinine (≥10 ng/mL) were considered reliable indicators for cigarette smoke.

^b^Low cotinine is defined as <10 ng/mL. Level for cotinine (<10 ng/mL) were considered reliable indicators for nonsmokers.

AC, atherogenic coefficient; BMI, body mass index; CHOL, total cholesterol; CRI-I, Castelli risk index-I; DBP, diastolic blood pressure; HbAlc, glycated haemoglobin; HDL-c, high-density lipoprotein cholesterol; LDL-c, low-density lipoprotein cholesterol; SBP, systolic blood pressure; TG, Triglycerides; WC, waist circumference.

Table 2 presents the association between serum cotinine (≥10 ng/mL) as a continuous variable and CHOL, HDL-c [log], LDL-c, TG [log], CRI-I [log], and AC [log] separately. Serum cotinine was positively associated with HDL-c [log] (*P* = 0.04), CRI-I [log] (*P* = 0.03), and AC [log] (*P* = 0.03) in the age, gender, waist circumference, diabetes medications, and statins-adjusted models. In contrast, there was no association between serum cotinine (as a continuous variable) and CHOL, LDL-c, and TG [log].

**TABLE 2:** Multiple linear regression analyses of the associations between serum cotinine (≥10 ng/mL) and lipid-related indices.

	Independent variable cotinine levels (≥10 ng/mL)^a^ *n* = 32				
Dependent variables	β	SE	*P*	*R* ^2^	*f* ^2^
CHOL^b^	0.001	0.002	0.40	0.12	−
HDL-c^b^ [log]	−0.001	0	0.04*	0.39	0.65**
LDL-c^b^	0.002	0.001	0.29	0.17	−
TG^b^ [log]	0	0.001	0.48	0.27	−
CRI-I^b^ [log]	0.001	0	0.03*	0.38	0.62**
AC^b^ [log]	0.001	0.001	0.03*	0.39	0.64**

*P is considered significant at <0.05. Bolded values mean statistically significant results.

^a^Level for cotinine (≥10 ng/mL) were considered reliable indicators for cigarette smoke.

^b^Dependent variables in separate multiple linear regression equations with covariates including age, diabetes medications, gender, statins, and waist circumference.

AC, atherogenic coefficient; β, regression coefficient; CHOL, total cholesterol; CRI-I, Castelli risk index-I; f^2^, Cohen’s effect size; HDL-c, high-density lipoprotein cholesterol; LDL-c, low-density lipoprotein cholesterol; R^2^, coefficient of determination; SE, standard error, TG, Triglycerides.

**f^2^ = R^2^/1-R^2^ calculated for significant regression models.

Table 3 presents the association between increasing quartiles of serum cotinine (≥10 ng/mL) and mean change in CHOL, HDL-c [log], LDL-c, TG [log], CRI-I [log], and AC [log], taking the lowest quartile of serum cotinine as the referent category (10 ≥cotinine ≤ 199 ng/ml). There was no association between increasing quartiles of serum cotinine and lipid-related indices. MLR indicated that there is some evidence for an effect, albeit rather weak evidence, for CRI-I [log] and AC [log] with *p*-values of 0.06 for both indices. We considered the conventional *p*-value of less than 0.05 to be statistically significant for all analyses, however, we reported the effect size and the 95% CI for each quartile. MLR results showed that participants in the 4th quartile (highest) of serum cotinine level, compared with those in the 1st quantile (lowest), had higher CRI-I (β = 0.35, 95% CI = −0.01, 0.72), and AC (β = 0.45, 95% CI = −0.02, 0.93) values. Participants in the 3rd quartile, compared with those in the 1st quartile, had higher CRI-I (β = 0.12, 95% CI = −0.23, 0.47) and AC (β = 0.16, 95% CI = −0.29, 0.62) values. Participants in the 2nd quartile, compared with those in the 1st quartile, had higher CRI-I (β = −0.02, 95% CI = −0.40, 0.35) and AC (β = −0.05, 95% CI = −0.54, 0.44).

**TABLE 3:** The associations of quartile of serum cotinine level (Reference: 10 ≥ cotinine ≤ 199 ng/ml) with lipid-related indices^a^.

	Quartile 1 (*n* = 8)	Quartile 2 (*n* = 8)	Quartile 3 (*n* = 8)	Quartile 4 (*n* = 8)
Dependent variables	10 ≥ cotinine ≤ 199 ng/ml	200 ≥ cotinine ≤ 324 ng/ml	325 ≥ cotinine ≤ 455 ng/ml	456 ≥ cotinine
CHOL*^a^*	Reference	β = −0.74, 95% CI = −2.32, 0.84	β = −0.24, 95% CI = −1.71, 1.24	β = 0.59, 95% CI = −0.94, 2.11
		(0.34)	(0.74)	(0.43)
HDL-c [log]^a^	Reference	β = −0.14, 95% CI = −0.45, 0.17	β = −0.19, 95% CI = −0.48, 0.10	β = −0.23, 95% CI = −0.53, 0.07
		(0.37)	(0.19)	(0.12)
LDL-c*^a^*	Reference	β = −0.48, 95% CI = −1.86, 0.90	β = 0.07, 95% CI = −1.22, 1.36	β = 0.53, 95% CI = −0.80, 1.86
		(0.48)	(0.91)	(0.42)
TG [log]*^a^*	Reference	β = −0.27, 95% CI = −0.77, 0.22	β = −0.16, 95% CI = −0.62, 0.31	β = 0.23, 95% CI = −0.25, 0.71
		(0.27)	(0.49)	(0.33)
CRI-I [log]*^a^*	Reference	β = −0.02, 95% CI = −0.40, 0.35	β = 0.12, 95% CI = −0.23, 0.47	β = 0.35, 95% CI = −0.01, 0.72
		(0.89)	(0.49)	(0.06)
AC [log]*^a^*	Reference	β = −0.05, 95% CI = −0.54, 0.44	β = 0.16, 95% CI = −0.29, 0.62	β = 0.45, 95% CI = −0.02, 0.93
		(0.82)	(0.47)	(0.06)

^a^Dependent variables in separate multiple linear regression equations with covariates including age, diabetes medications, gender, statins, and waist circumference.

AC, atherogenic coefficient; β, regression coefficient; CHOL, total cholesterol; CI, confidence interval; CRI-I, Castelli risk index-I; HDL-c, high-density lipoprotein cholesterol; LDL-c, low-density lipoprotein cholesterol; TG, Triglycerides.

## Discussion

Cigarette smoking is associated with several CVDs, including atherosclerosis, heart failure, and stroke all of which result in damage to the heart and blood vessels. Active smoking may result in anatomical and biochemical changes to the coronary arteries and the myocardium, consequently leading to CVD. Examining a variety of diagnostic biomarkers that potentially assesses changes to components of the cardiovascular system may be beneficial in CVD management. Cigarette smoke contains nicotine in considerable concentration. Cotinine, a major metabolite of nicotine, is a specific and highly representative parameter for exposure to cigarette smoke. The chronic effects of cotinine on the cardiovascular system include unfavourable effects on specific lipoprotein levels, which play a role in the initiation of CVD (29). Based on findings of this study, it is suggested that serum cotinine be added to the standard of care for T2D management as it may provide important insights to lipid abnormalities and CVD risk among Turkish immigrants with T2D.

The present study showed that lipid ratios of HDL-c, CRI-I, and AC are positively associated with serum cotinine levels (≥10 ng/mL). These findings corroborate other studies where there is a significant association in HDL-c levels in smokers than in non-smokers and an association between inhaled cigarette smoke and the atherosclerotic risk (27, 30, 31). Our study evaluated lipid ratios of CRI-I and AC since these atherogenic indices are validated parameters associated with increased CVD risk. A cross-sectional study with 699 participants indicated that CRI-I predict the highest prevalence of predisposition to CVD risk (47.8%) (27). The Castelli risk index-II (CRI-II), atherogenic index of plasma (AIP), and CHOLIndex have been proposed as new lipid parameters in assessing CVD risk (27, 32). Olamoyegun et al. (27) reported that the AC, CRI-II, CHOLIndex, and AIP predicted a CVD risk prevalence of 22.5, 15.9, 11.2, and 11.0%, respectively. Some research suggest that alternative lipid-related indices be used to evaluate CVD risk even when serum lipid levels seem normal (32). According to the Framingham Heart Study (30), smokers who quit for more than 1 year have blood cholesterol levels similar to non-smokers. Garrison et al. (30) suggest that for males, a CRI-I of 5 signifies average risk for CVD. Whereas, females tend to have higher HDL-c levels; therefore, a ratio of 4.4 signifies average risk. This suggests that a high level of CHOL may be less alarming if the CRI-I ratio is low. Interestingly, studies have shown that Turkish immigrants tend to have a lower prevalence of high CHOL levels, a known risk factor for CVD, as compared to the native Dutch (9, 13, 14, 17, 33). Results of our study are corroborated by previous studies that showed low CHOL levels among the Turkish immigrant population, regardless of statin use.

Patients with T2D are more prone to dyslipidaemia, a major risk factor for CVD in T2D. Diabetes-induced dyslipidaemia may be manifested by reduced HDL-c, increased LDL-c, and increased TG levels in the blood (11, 34). In patients with diabetic dyslipidaemia, statin therapy has beneficial effects to cardiovascular health (11). However, other than decreasing the LDL-c levels, the management of dyslipidaemia in patients with T2D continues to be challenging (35). Another factor that may increase risk of dyslipidaemia includes cigarette smoking. Reiss et al. (36) conducted a systematic review to examine the smoking prevalence among immigrant population from non-Western and Western countries. The majority of the studies (23 of 27) reported a strong association between gender and smoking behaviour. Socio-demographic factors including age, gender, and education are known to determine smoking behaviour across all population groups. Nierkens et al. (15) investigated the smoking prevalence among three immigrant populations in the Netherlands and results show that the prevalence of smoking was highest among the Turkish, with 63% of Turkish males who smoke and 32% of Turkish females who smoke. Additionally, the youngest immigrant population (34–44 years) had higher smoking rates as compared to the older age group (45–60 years). Educational level in relation to smoking behaviour have been examined in several studies and results show that Turkish males with a lower education and Turkish females with a higher education tend to have higher smoking rates (13, 15). Our study revealed that education was significantly associated with high serum cotinine levels (*P* = 0.02).

There is a high prevalence of smoking among Turkish in country of origin (Türkiye) and host country (Netherlands) (36). Cultural perceptions of smokers among Turkish immigrants warrant further investigation as the high rate of smokers may be influenced by cultural practices (13, 37). Furthermore, environmental determinants of smoking including living in deprived neighbourhoods and socioeconomic status (SES) are known to adversely impact health (15, 17, 22). Middelkoop et al. (38) characterised The Hague by the largest variation in neighbourhood deprivation scores and highest level of segregation in the Netherlands. Mortality data of residents, under the age of 65 years, show that CVD was responsible for cause of death among 32.5% males and 21.3% females living in The Hague (38). Data indicate that mortality risk increased in deprived neighbourhoods, and a strong association between deprivation score and percentage of non-Western inhabitants. A recent study conducted in the Netherlands emphasises the importance of GP awareness to reach smokers in deprived neighbourhoods (22). The prevalence of GP healthcare services is generally observed to be much higher among Turkish in the Netherlands (9, 13). Turkish tend to report more complaints to the GP, are more likely to seek advice from a specialist, and have high hospital-admission rates with regards to heart complaints (13). In our study, the majority of participants (54.5%) were recruited from GP offices. Based on previous studies, it is suggested that GPs may play an important role in effective T2D management (39–42). Health professionals are encouraged to develop ethnically targetted interventions in the Netherlands (17, 33, 36, 39, 43–45). Approximately 10% of the population in the Netherlands are non-Western immigrants, which is expected to increase to 20% by 2060 (43). Interestingly, non-Western immigrants have a lower average SES than the native Dutch population (43). The health effects of SES are determined by several risk factors including physical living (neighbourhood deprivation) and health-related behaviour (cigarette smoking). Effective preventive and treatment approaches to T2D management among Turkish immigrants may decrease the burden of ethnic inequalities in health (33, 39, 45). Ethnic-specific data could help GPs assess which indicators to be added to the standard of care.

This study examined first-generation Turkish immigrants with T2D living in a deprived neighbourhood of The Hague, Netherlands. Turkish form the largest first-generation immigrant group in the Netherlands, with a large population living in the most deprived neighbourhoods (14, 17). Research has shown that ethnic minority groups are less likely to participate in research studies, possibly due to language barrier and low literacy. Despite limitations such as these, this study adds to the scarce research in a poorly represented group. It is suggested that non-Western immigrants may import their smoking behaviour from the country of origin to the host country. However, Reiss et al. (46) report that immigrants themselves could potentially influence the majority population’s health behaviour. Environmental stressors including living in deprived areas may also increase smoking behaviour. A longitudinal study conducted in the Netherlands indicates a causal relationship between neighbourhood deprivation and smoking status (47). The authors conclude that the socioeconomic characteristics of areas in which smokers reside impact their quitting behaviour. According to the CDC–Office on Smoking and Health (48), several studies suggest an association between cigarette smoking and low SES. The 2015 CDC Morbidity and Mortality Weekly Report (49) states that low SES populations are at an increased risk of SHS exposure and therefore prone to being affected from the harmful effect of cigarette smoke. In the aforementioned study, SHS exposure was determined using serum cotinine; the primary metabolite of nicotine. The CDC–National Biomonitoring Programme (25) currently recommends cotinine as the best biomarker of ascertaining nicotine exposure.

In large nationally representative samples of US adults (NHANES 2003–2012), higher serum cotinine levels are associated with risk of developing diabetes among never smokers exposed to SHS (50). Additionally, previous studies consistently show that risk factors including T2D, cigarette smoking, and dyslipidaemia are associated with CVD morbidity and mortality (10–12, 51). Lifestyle modification including smoking cessation could potentially provide some benefit in high-risk populations with diabetes-induced dyslipidaemia. To the best of our knowledge, this is the first study to examine the association of serum cotinine and lipid-related indices among first-generation (foreign born) Turkish immigrants with T2D living in deprived neighbourhoods of The Hague, Netherlands. Diabetes-induced dyslipidaemia is a known risk factor for CVD; therefore, examining modifiable risk factors such as cigarette smoke could provide important insights into the possible role of successful T2D management. This study demonstrated that cigarette smoke, based on serum cotinine ≥10 ng/mL, had a negative effect on specific lipid-related ratios. Cotinine is the main metabolite of nicotine. and preferred biomarker in clinical studies, since it is not affected by the environment or diet, thus completing requirements of specificity and half-life in the body (52). Raja et al. (53) reported that serum cotinine has a longer half-life, as compared to salivary cotinine, and does not need adjusting for hydration difference among individuals as in saliva testing. A prospective study done in the Netherlands concluded that serum cotinine showed greater stability than urinary cotinine and is therefore more practical for use in clinical settings (54). The scientific literature report varying cut-off points for serum cotinine levels (25, 50). The Society for Research on Nicotine and Tobacco (SRNT) Subcommittee on Biochemical Verification (55), report that cotinine levels range from 10 to 20 ng/ml due to variability in diverse racial groups. We determined a serum cotinine cut-off value of ≥10 ng/mL to be an indicator of active smoking, as recommended by the CDC–National Biomonitoring Programme (25).

Wakabayashi (31) found that in patients with diabetes, the levels of lipid-related indices were higher in smokers than in non-smokers, which plays a major role in the development of atherosclerotic CVD. While the prevalence of smoking is high in Turkish immigrants (13–15), few studies have examined the effect of serum cotinine and its role in T2D and CVD in this high-risk population. Dyslipidaemia coupled with T2D and cigarette smoking could potentially lead to the high CVD prevalence observed among Turkish immigrants living in the Netherlands. An observational study done in Türkiye among 307 patients who underwent diagnostic coronary angiography found the CHOLIndex to have an independent predictive value for CVD (OR = 1.011, *P* = 0.009) (32). Akpınar et al. (32) indicated that age, diabetes status, male gender, and cigarette smoking were independent predictors of CVD. Furthermore, the authors suggest the CHOLIndex appropriate when evaluating lipid-related risk for CVD. Alternative ethnic-specific lipid and lipoprotein parameters should be investigated in larger cohorts with T2D to assess the association of serum cotinine, as an indicator of active and passive cigarette smoke exposure, and CVD risk.

The high prevalence of smoking among Turkish immigrants in the Netherlands poses a major public health concern. Smoking habits of active smokers could potentially endanger others to SHS. Recent data from large nationally representative samples of US adults show an association between higher serum cotinine levels and associated diabetes among never smokers (50). Additionally, Jain and Ducatman’s study (18) concluded that serum cotinine was significantly associated with unfavourable lipid/lipoprotein profiles among the adult population of the USA. Modification of lifestyle risk factors that includes prevention of cigarette smoke could potentially lessen CVD risk in Turkish immigrants with and without T2D. A comparative analysis of 6,517 Turkish immigrants showed a higher smoking prevalence among Turkish immigrants in the Netherlands than among their counterparts in Germany (46). Data from the LASER study reported the highest prevalence of smoking in first-generation Turkish males (54.9%) as compared to second-generation Turkish males (45.6%) and native Dutch males (36.2%) (16).

The high prevalence of T2D among Turkish immigrants in the Netherlands remains unclear. There is a much higher rate of T2D observed in Türkiye when compared to the Netherlands (9). Studies indicate that the diets of non-Western immigrants differ from that of the native Dutch (13, 56). van Leest et al. (13) report that Turkish immigrants tend to eat more fruits and vegetables. However, there is a high prevalence of overweight and obesity among first-generation Turkish immigrants in the Netherlands (20). Physical activity should also be considered when assessing serum levels. Research shows that Turkish immigrants are less likely to be physically inactive, especially first-generation immigrants (≈10%) (13). Genetics also plays an important role in the low levels of HDL-c typically observed among the Turkish population (29). Clinical practice guidelines and adherence for T2D management in Dutch GP offices can be challenging (40, 42). The 2013 NHG standard for T2D and 2012 NHG standard for CVD risk management recommend that patients with modifiable risk factors, should be given the following lifestyle advice to reduce the risk of CVD: no smoking, optimal physical activity, healthy diet, optimum weight, and avoidance of stress (41, 42).

The pathogenesis of CVD in T2D is multifactorial and cigarette smoking and dyslipidaemia are known to be powerful independent risk factors. The detrimental health effects of cigarette smoke are well documented and have been linked to many chronic diseases. The 2014 Surgeon General’s Report on smoking and health, states that smoking is a major cause of CVD and causes one of every three deaths from CVD (57). Additionally, the relationship between carbohydrates and lipid metabolism in patients with T2D may be affected by various factors. High-risk populations with T2D are more prone to dyslipidaemia since insulin resistance disrupts important enzymes and pathways in lipid metabolism. Numerous studies indicate that lipid-related indices are good indicators to assess CVD risk in patients with T2D (27, 30–32). Among the indices examined, results of this study show that cigarette smoke as defined by high serum cotinine levels has better predictability to assess lipid ratios of HLD-c, CRI-I, and AC in Turkish immigrants with T2D living in deprived neighbourhoods of The Hague, Netherlands. However, there is a need for additional lipid-related indices to be measured in larger cohorts that include prospective studies to assess serum cotinine levels and CVD in high-risk populations with diabetes and lipid irregularities.

### Strengths and limitations

This study adds to the body of research on serum cotinine, as an indicator for cigarette smoke, and lipid irregularities in high-risk populations with T2D. de Weerd et al. (54) suggest serum cotinine to be a more reliable measure, as opposed to urinary cotinine, for use in clinical settings. A major strength of this study is that serum cotinine was examined to minimise the potential for misclassification of smoking status and to emphasise biochemical confirmation of cigarette smoke. The participants were asked not to change their smoking behaviours on the day of blood collection. Participants included are all of Turkish descent, a single origin, in which research concerning serum cotinine and lipid abnormalities in T2D are limited. Turkish immigrants were examined because of the high prevalence of CVD, cigarette smoking, and T2D when compared to the native Dutch (9, 13–15). We used standardised protocols, including uniform anthropometric and biochemical measurements in a clinical setting. We adjusted the statistical analysis for all major confounders of cotinine and lipids including age, gender, WC, diabetes medications, and statins. One strength of this study is that due to the availability of Turkish-speaking staff, also those first-generation Turkish immigrants who cannot read or write, and are not able to understand or speak Dutch, were included. Our study investigates modifiable health-risk behaviours, such as cigarette smoking, in a vulnerable population living in deprived neighbourhoods of The Hague, Netherlands. Neighbourhood characteristics affect ethnic inequalities in health, an area where more research is needed to confirm our preliminary findings. Another major strength of this study is that our cross-sectional design was conducted according to the Strengthening the Reporting of Observational Studies in Epidemiology (STROBE)-guidelines (58).

There are a number of potential limitations in this study. Due to the study’s cross-sectional nature, our results do not establish causality and cannot be generalised to other populations. Several studies indicate that metformin and statins significantly reduce CHOL, LDL-c, and TG, and increase HDL-c levels. Additional statistical analyses were done to determine the potential confounding effect of diabetes medications and statins. Results of these tests verified that the direction did not change. All MLR models (Tables 2, 3) were adjusted for the aforementioned covariates. Further statistical analyses were done to determine the potential effect size and the 95% CI for each quartile of higher serum cotinine level by taking the lowest category as the referent (10 ≥cotinine ≤199 ng/ml). Our findings did not indicate a significant association between increasing quartiles of serum cotinine and lipid-related indices.

Although serum cotinine and lipid levels are ethnically specific, our sample was not randomly selected and may not represent the Turkish immigrant population of the Netherlands. Longitudinal studies are recommended to determine the effect of serum cotinine on lipid-related indices and consequent CVD morbidity and mortality in Turkish immigrants with T2D. The study sample size (*n* = 110) also warrants further investigation in larger cohorts. However, the sample size of our study is comparable to other studies in the Netherlands (17, 22, 43, 44). El Fakiri et al. (17) investigated the prevalence of cardiovascular risk factors among various ethnic groups at high risk of developing CVD within GP offices in the Netherlands. The researchers analysed data on 126 Turkish patients, among other ethnic groups (17). Benson et al. (22) investigated smoking cessation behavioural therapy among 59 ethnic minorities, including Turkish, living in disadvantaged neighbourhoods of the Netherlands. Schouten et al. (44) analysed data of the Rotterdam Intercultural Communication in Medical Settings (RICIM) project, in which patients of 38 GP offices with a multi-ethnic population participated. The RICIM dataset investigated 103 patients (56 patients belonging to one of the major ethnic minority groups in the Netherlands—Turkish, Moroccan, Surinamese, Antillean, Cape Verdian—and 47 Dutch patients). Our research study collected data within a 3-month period from a high-risk first-generation Turkish immigrant population living in a deprived neighbourhood of The Hague, Netherlands, where research is limited. Recruitment methods support other studies that indicate that GPs are most effective in reaching the immigrant population, specifically when recruiting smokers living in disadvantaged areas (59–61).

## Conclusion

This cross-sectional study identifies the compelling need to investigate several important clinical and public health concerns related to smoking and unfavourable lipid/lipoprotein profiles in Turkish immigrants with T2D, preferably by well-designed longitudinal prospective studies and randomised clinical trials. It is well-known that T2D is a multidimensional disorder and there is an increasing need to address the biomedical root causes of CVD risk and the adverse effect induced by lipid/lipoprotein markers. These should be looked at as aetiological causes of CVD risk in patients with T2D. Additionally, the pathogenesis of T2D and CVD are associated with elevated lipid/lipoprotein markers such as CHOL, LDL-c, HDL-c, and TG (18). The present study indicates that lipid ratios of HDL-c [log], CRI-I [log], and AC [log] are dependent determinants of serum cotinine and higher serum cotinine levels (≥10 ng/mL) are associated with worse HDL-c [log], CRI-I [log], and AC [log] values in participants with T2D. Clinical comprehension of these biochemical indicators (lipids/lipoproteins) and symptomatic results (CVD risk) in patients with T2D will aid in the intervention (smoking) approach for this vulnerable cohort (Turkish immigrants).

Smoking is a preventable risk factor for CVD and public health awareness of its adverse effects in individuals with T2D should be promoted among high-risk populations. Prevention and decrease of cigarette smoke in Turkish immigrants with T2D may help protect their cardiovascular health and decrease CVD risk. Adding serum cotinine testing to the standard of care for T2D may direct management in the primary prevention of CVD among first-generation Turkish immigrants. There is a need for vulnerable groups living in deprived neighbourhoods to be further educated on effective T2D self-management. Therapy that is targetted to modify this behavioural risk factor may improve cardiovascular health outcomes and prevent comorbidities in Turkish immigrants living in deprived neighbourhoods in the Netherlands. In the meantime, this report contributes to a growing body of information and provides essential guidance to researchers and clinicians.

## Data availability statement

The raw data supporting the conclusions of this article will be made available by the authors, without undue reservation.

## Ethics statement

The studies involving human participants were reviewed and approved by (1) Central Committee on Research Involving Human Subjects; (2) Florida International University Institutional Review Board (project identification code: 111210-01, date of approval: 12 February 2010). Informed consent was obtained from all individual participants included in the study. The patients/participants provided their written informed consent to participate in this study.

## Author contributions

SS designed and coordinated the study, participated in data collection and analysis, and drafted the manuscript. GZ prepared the original data files, performed the data analysis, and was involved in critical revision of the manuscript. LS contributed to the design of the study and critical revision of the manuscript. JV contributed to the interpretation of the data and helped to bring the manuscript to its final version. AS contributed to the acquisition and interpretation of data and helped to finalize the manuscript. FH contributed to the design and coordination of the study and critically revised the manuscript. All authors read and approved the final manuscript.
