# Supplementary figures and images for: Serum cotinine as a predictor of lipid-related indices in Turkish immigrants with type 2 diabetes: A clinic-based cross-sectional study
Source: Front Med (Lausanne). 2023 Feb 16;10:1011045. doi: 10.3389/fmed.2023.1011045 (PMC9978330; doi:10.3389/fmed.2023.1011045)

**Supplementary Figure 1.** Study enrolment flow chart.

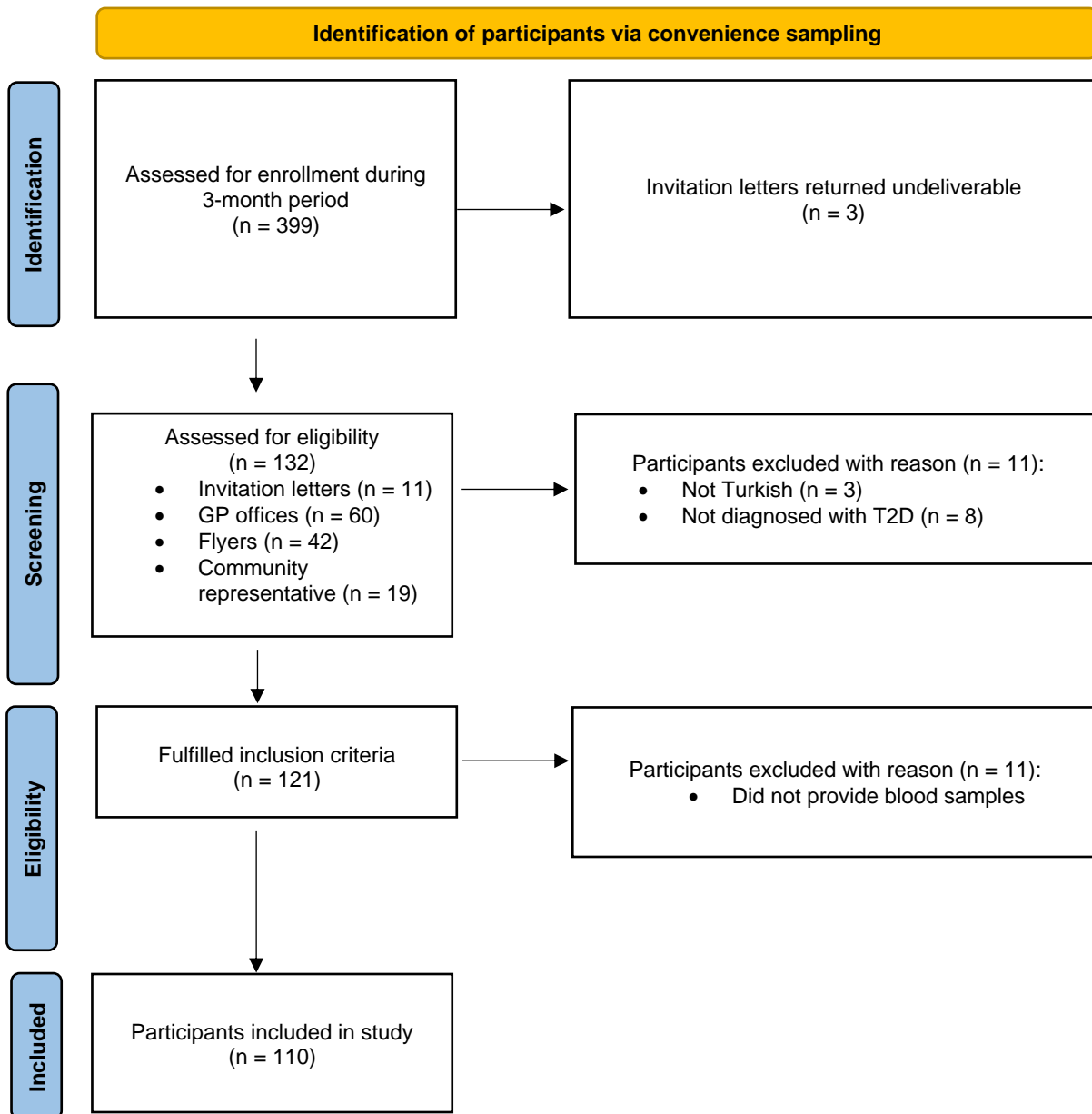

Supplement: Supplementary file 1 [file Image_1.pdf]
